# Data-Driven Dietary Patterns, Nutrient Intake and Body Weight Status in a Cross-Section of Singaporean Children Aged 6–12 Years

**DOI:** 10.3390/nu13041335

**Published:** 2021-04-17

**Authors:** Michelle Jie Ying Choy, Iain Brownlee, Aoife Marie Murphy

**Affiliations:** 1 Devan Nair Building, Newcastle Research and Innovation Institute, Newcastle University, Singapore 600201, Singapore; michelle.choy@outlook.com (M.J.Y.C.); aoife.murphy.4@ucdconnect.ie (A.M.M.); 2 Faculty of Health and Life Sciences, Northumbria University, Newcastle-upon-Tyne NE1 8ST, UK

**Keywords:** dietary intake, cluster analysis, dietary pattern, dietary recall

## Abstract

Pattern analysis of children’s diet may provide insights into chronic disease risk in adolescence and adulthood. This study aimed to assess dietary patterns of young Singaporean children using cluster analysis. An existing dataset included 15,820 items consumed by 561 participants (aged 6–12 years) over 2 days of dietary recall. Thirty-seven food groups were defined and expressed as a percentage contribution of total energy. Dietary patterns were identified using *k*-means cluster analysis. Three clusters were identified, “Western”, “Convenience” and “Local/hawker”, none of which were defined by more prudent dietary choices. The “Convenience” cluster group had the lowest total energy intake (mean 85.8 ± SD 25.3% of Average Requirement for Energy) compared to the other groups (95.4 ± 25.9% for “Western” and 93.4 ± 25.3% for “Local/hawker”, *p* < 0.001) but also had the lowest calcium intake (66.3 ± 34.7% of Recommended Dietary Allowance), similar to intake in the “Local/hawker” group (69.5 ± 38.9%) but less than the “Western” group (82.8 ± 36.1%, *p* < 0.001). These findings highlight the need for longitudinal analysis of dietary habit in younger Singaporeans in order to better define public health messaging targeted at reducing risk of major noncommunicable disease.

## 1. Introduction

The study of diet–disease relationships has been previously conducted through the examination of individual nutrients or food groups and their relationship with risk factors or outcomes of chronic diseases [1,2]. However, overall dietary habit often comprises of a diverse range of inter-related food items providing a complex combination of nutrients and food group intakes, which may not be adequately modelled through the single-nutrient or single-food approach [3,4]. Results from human intervention studies have shown positive health outcomes with changes in multiple facets of dietary behaviour [5,6]. “A posteriori” dietary patterns” is a term that refers to data-driven commonalities in estimated habitual food intake [7]. When such patterns are noted within populations, they often highlight divergences in overall nutrient intake and/or markers of nutritional status [4,8].

While a posteriori dietary patterns have been widely investigated in Western and European countries [9,10,11], relatively few studies have been conducted in Asian countries [12,13]. As a multicultural hub of trade, Singapore has many culinary influences [14] and imports foods from most regions of the world [15]. Longitudinal changes to population-level dietary habit as a result of Singapore’s rapid increase in affluence that have occurred in parallel with increased prevalence of cardiometabolic health issues, particularly type II diabetes [16]. While nationally representative dietary intake data have been collected from adults aged 18–69 year olds since 1993 [17], estimation of dietary habit in older or younger Singaporeans is limited. Previous studies in adult Singaporean populations have highlighted that more prudent a posteriori dietary patterns were associated with reduced risk of major health issues [18,19,20,21].

The prevalence of overweight and obesity among Singaporean children has increased over the past three decades [22,23], highlighting a need for understanding of existing dietary habits in this population. Previous longitudinal studies have associated poor dietary patterns in children with the onset of chronic diseases in adolescence and adulthood [11,24,25], further underlining the need for additional evaluation of dietary habits in young Singaporeans. A recent study collected cross-sectional data on dietary habit in 6–12-year-old Singaporean children [26], providing a unique dataset to evaluate for the presence of dietary patterns within this age group. The present study aimed to (i) assess dietary patterns of children aged 6–12 years using cluster analysis (ii) evaluate the dietary patterns derived from this a posteriori method in terms of estimated nutrient provision and (iii) consider how body mass index (BMI) and demographic factors were associated with such patterns.

## 2. Materials and Methods

### 2.1. Study Population

Data used in this project was obtained from a study conducted by Neo et al. in 2016 involving a nationally representative cross-section of 561 6–12 year olds (52% female, 15% overweight or obese), further detail of which is available in the original study [26]. This cross section was comprised of 14% 6 year olds, 53% aged 7–9 and other aged 10 and above. Briefly, dietary data were collected between April 2014 and January 2015 in Singaporean children through duplicate 24 h food recalls completed by both child and parent during home visits (ethical approval granted on the 24th January 2014 by Newcastle University Science, Agriculture, and Engineering Ethics Committee–14-BRO-053). Data were collected for the previous day’s dietary intake by trained researchers using the multiple pass method, with visual aids to support estimation of food portion size [26]. Height and weight were measured (using portable weighing scales and stadiometers) during these visits to calculate participant BMI and demographic information was also collected by questionnaire. BMI category was defined based on existing Health Promotion Board BMI age for percentile charts [27,28]. Age for each participant was taken as their age in years up at the point of the first data collection visit. Dietary data were analysed using WinDiets Professional 2015 software (Robert Gordon University, Aberdeen, UK), then compiled with other study data. These anonymised data were used within the current study.

### 2.2. Dietary Data Analysis

The cluster analysis method uses multivariate statistical techniques, where large datasets representing total food intake are aggregated and reduced to smaller datasets to summarise estimated total dietary exposure [29]. Cluster analysis gathers individuals into non-overlapping groups (clusters) based on similarities between their diets.

A total of 15,820 food items were recorded from the multiple 24 h dietary recalls in the original dataset. All food items consumed were sorted into one of 65 food groups which were determined according to the Health Promotion Board’s food group classification through their online nutrient analysis tool, “Energy and Nutrient Composition of Foods” which provides the energy and nutrient content of popular local and western foods and beverages, as well as popular fast foods [30]. The 65 food groups were further aggregated into 37 food groups according to their nutrient profiles and on commonly identified dietary pattern analysis themes. For example, the sub-groups “beef and veal” and “mutton and lamb” were combined into a single food group and named as “red meat”. Modifications were made to the food group “beverages”, where the original sub-groups were “alcoholic beverages” and “non-alcoholic beverages” and were not rational to include in a sample who consumed no alcoholic drinks over the data collection period. In part due to public health concerns around diabetes in Singapore, sugar-sweetened beverages and their impact on health have become of particular interest [31]. As such, all beverages were classified as “sugar-sweetened beverages” and “other beverages” (all food outlet and manufactured beverages without any added sugars). The list of 37 food groups which are representative of the overall diet can be found in Supplementary Table S1.

Dietary data were standardized against recommended intake values defined by age, sex and physical activity using a similar approach applied to evaluation of infant dietary habits in a previous study [32]. Wherever possible, these were compared to Recommended Dietary Allowance values used in Singapore [33]. Where no national guideline existed for Singaporean children the United States Department for Agriculture (USDA) Dietary Guidelines for Americans 2020–2025 were used [34]. Data for energy and micronutrient intake are presented as percentages of these recommended intake levels.

### 2.3. Identification of Dietary Patterns

Data processing was carried out using SPSS v25 software (IBM SPSS Inc., Chicago, IL, USA). Original dietary recall data was collected on one weekday and one weekend day.

The mean daily nutrient intake of each food group (grams per day) was calculated for each individual. This first involved defining estimated daily intake data for all nutrients from each food group for each participant. The sum of the daily intake of each nutrient for the weekday and weekend day was divided by 2 (2-days food recall) to calculate the mean daily nutrient intake per participant.

Estimates of energy and nutrient intake were made as previously described outcomes of dietary intake analysis [26,35]. The cluster analysis was performed using the k-means algorithm which provides a measure of Euclidean distance from each record to the cluster centre and from each cluster to the others [9]. Clusters with smaller distances between one another have a greater degree of similarity while clusters with larger distances are more divergent [36]. The first step of cluster analysis suggested a 3-cluster solution. Further analyses were used to evaluate cluster proximities for each cluster centre to ensure minimum error in cluster membership. Therefore, further runs of 2- and 4-cluster solutions were also conducted. The best cluster solution (the 3-cluster solution) was selected based on the degree of variation in the sample which was elucidated by the size of the cluster, ease of interpretation and reliability of the cluster membership. Each cluster was then named according to a dietary pattern that was associated with the food groups that were representative of each cluster.

### 2.4. Statistical Analyses

The SPSS® statistical software package v25 (SPSS Inc., Chicago, IL, USA) was used for data manipulation, k-means cluster analysis and basic statistical analyses of the data sets. Chi-Square tests were used to compare the distribution of participants by sex, age and BMI in relation to the different dietary patterns among the entire cohort and the three dietary patterns derived from cluster analysis, as well as the proportion of individuals meeting national recommendations for nutrient intake. Differences in the mean percentage contribution of each food group across clusters, mean nutrient intake across clusters, and cohort characteristics across clusters were evaluated using one-way analysis of variance (ANOVA). For statistically significant results (*p* < 0.05), further comparisons of the means were evaluated using post hoc Bonferroni tests. The confidence level of statistical analyses was set at 95%, where *p* < 0.05 was considered to show significant differences in mean values.

## 3. Results

### 3.1. Dietary Patterns in Singaporean Children by Cluster Analysis

The three-cluster solution was derived from percentage total energy contribution per food group (see Table 1). The three clusters were labelled as “Western”, “Convenience” and “Local/Hawker” type of foods.

Cluster 1: “Western”—this was the most prevalent cluster, noted in 42.6% of the sample population. This cluster is heavily represented by “biscuits, cakes and pastries”, “breads and rolls” and “leafy and non-leafy vegetables”, while also having moderately higher “red meat”, “stock cubes and yeast extracts”, “cheeses” and “manufactured soups”.

Cluster 2: “Convenience”—this cluster is represented by “local cakes, desserts and snacks”, “nuts and seeds, pulses and products”, “milks”, with somewhat higher “breakfast cereals” and “sugars, sweets and confectionery”.

Cluster 3: “Local/Hawker”—this cluster was the least prevalent cluster apparent in 19.8% of the sample population. This cluster is particularly represented by “grains and noodles” and “sweetened beverages”, as well as “fast foods” and “white meat”.

### 3.2. Demographic and Anthropometric Characteristics across Clustered Dietary Patterns

There was a slightly higher percentage of girls to boys in the “Western food” (51:49) and “Convenience food” clusters (55:45), although this was not statistically different (*p* = 0.668) from the equal ratio (50:50) in the “Local/Hawker food” cluster. The proportion of kindergarten (i.e., 6-year-old) children, lower primary children (i.e., 7–9 year olds and upper primary children (i.e., 10–12 year olds) was not statistically different across all dietary clusters (% Kindergarten: % Lower Primary: % Upper Primary = 18:51:31 for “Western”, 11:54:35 for “Convenience” and 13:56:32 for “Local/Hawker”, *p* = 0.150). The proportion of children who were underweight, acceptable weight and overweight by Health Promotion Board classifications were also similar across the three food clusters (% underweight: % acceptable weight: % overweight = 9:78:13 for “Western”, 9:76:15 for “Convenience” and 9:74:17 for “Local/Hawker”, *p* = 0.726).

### 3.3. Nutrient and Energy Intake for Each Dietary Cluster

The average nutrient and energy intake in relation to recommendations or proportion of total energy within each of the three clustered groups is presented in Table 2. The participants who were aligned with the “Western” and “Local/Hawker” clusters had a statistically higher (*p* < 0.001) mean ± SD % of recommended energy intake (95.4 ± 25.9% and 93.4 ± 25.3% Average Requirement for Energy, respectively) compared to the “Convenience” cluster group (85.8 ± 25.3% Average Requirement for Energy).

The “Western” cluster group also tended to have statistically higher (*p* < 0.001) mean intake of calcium (82.8 ± 36.1% of age-specific Recommended Dietary Allowance) than the “Local/Hawker” and “Convenience” groups (69.5 ± 38.9% and 66.3 ± 34.7% of age-specific Recommended Dietary Allowance, respectively). The % of energy consumed as fat and saturated fat was lowest (*p* < 0.001) in the “Convenience” group (28.3 ± 5.4% and 7.6 ± 2.8%) in comparison to the “Western” (30.7 ± 5.9% and 8.3 ± 2.9%) and “Local/Hawker” groups (31.7 ± 7.1% and 9.1 ± 3.4%). The mean percentage of dietary energy consumed as protein was different across all groups (16.5 ± 3.4% in the “Western”, 17.3 ± 3.5 “Convenience” and 15.3 ± 3.6% “Local/Hawker” groups, respectively), with around 95% of all participants consuming adequate amounts of dietary protein by age and sex (data not shown). Although one-way ANOVA tests presented *p*-values suggestive of differences between groups in relation to the % of energy from carbohydrates and added sugars (*p* = 0.047 and 0.040, respectively), targeted post hoc tests suggested no statistical difference between each group (*p* ≥ 0.056 and for %E from carbohydrates and *p* ≥ 0.074 %E from added sugars for comparisons across cluster groups).

Vitamin D intake appeared to be frequently low across dietary pattern groups (27.1 ± 67.8, 25.5 ± 59.2 and 18.7 ± 48.7% of Recommended Dietary Allowance across “Western”, “Convenience” and “Local/Hawker” groups, respectively, *p* = 0.479), with only 6.25% of participants meeting recommendations for their age and sex). Average calcium intake tended to align more closely with recommendations in the “Western” group (*p* < 0.001, 82.8 ± 36.1% of Recommended Dietary Allowance) in comparison to “Convenience” (66.3 ± 34.7%) and “Local-Hawker” (69.5 ± 38.9%) groups.

## 4. Discussion

To the authors’ knowledge, this study is the first attempt at using a posteriori approaches to evaluate dietary patterns among Singaporean children aged 6 to 12 years old. The authors feel that a cluster analysis is an effective approach to model the underlying patterns and dietary diversity that might be expected in a multi-ethnic, multicultural food centre like Singapore [37].

The three dietary patterns derived from cluster analysis reflected the common Singaporean practice of eating out of home or buying takeaways. A commonality across all three clusters is that many items were purchased and/or consumed outside of the home. A previous report noted that one-fifth of children and adolescents ate out of home 5 to 7 times a week due to convenience and lack of homecooked meals by parents or caregivers [38]. Previous studies have suggested that k-means cluster analysis has a considerable advantage over Principal Component Analysis (another common approach to dietary pattern analysis) in terms of allowing a clearer separation of sub-groups by dietary pattern [39,40,41].

These findings broadly align with the previous a priori evaluation of dietary habit that highlighted frequent low intake of fruits, vegetables and whole grains within this cohort [35]. All dietary pattern groups appeared to have similarly low average levels of dietary fibre consumption, which further aligns with these previous findings. Each of the three clusters represented dietary patterns of concern, including many processed food items. No cluster identified prudent dietary patterns. Comparisons of nutrient intake between groups suggested that each dietary cluster was characterised by at least one food groups that it is recommended to be consumed in moderation. For example, baked products and red meat were noted in the “Western” dietary pattern, local desserts and snacks, sweets and confectionary commonplace in the “Convenience” dietary pattern, while more frequent sweetened beverages and processed food intake was noted in the “Local/hawker” dietary pattern. Such food items tend to result in higher total fat, saturated fat, added sugars and sodium intake, and tend to have more limited contributions to dietary fibre, vitamins and most minerals [13,25]. While nutrient deficiency in Singaporean children is not reported to be an overt issue, previous work has highlighted the rapid increase in prevalence of obesity, type II diabetes and other non-communicable diseases in Singapore [16]. Recently developed public health interventions aim to improve dietary intake in the school setting and outside of the home to help improve lifelong dietary habits in Singaporeans [42,43]. The current findings underline the need for such interventions and further support a need to engage the wider family unit in developing more positive dietary habits in children.

The “Local/hawker” dietary pattern was also associated with limited intake of fruit and vegetables, with most of the food items in the ‘grains and noodles’ food category, the majority of which were refined rather than wholegrain products. All groups also had average estimated intakes of sodium and added sugar that were above national Recommended Dietary Allowances [33]. These findings on high sodium intake somewhat align with those of the previous Student Health Survey (carried out from 2009 to 2012) which suggested that intentional addition of salt and high-salt condiments to food was common (12.7% and 19.4%, respectively) within Singaporean school children [44].

While average calcium intake was highest in the “Western” dietary pattern group, intake did not meet national Recommended Dietary Allowances [33] within this cross-section. Vitamin D intake was also frequently sub-optimal while the majority of participants appeared to consume adequate protein and iron. On their own, these findings highlight the potential for increased risk of bone health issues in Singaporean children, in whom developing peak bone mass is crucial to reduce the risk of osteoporosis in later life [45]. A previous cross-sectional study suggested that vitamin D status was sub-optimal in around 4 out of ten Singaporean adults [46], underlining that both sun avoidance and low levels of dietary vitamin D intake appeared to be commonplace. A recent study in older Singaporeans also suggested that over half of those interviewed were at risk of osteoporosis, a proportion which would be expected to rise with a rapid move towards an older population demographic [47]. However, findings from the a priori evaluation of dietary habit in this cohort suggested that most (>75%) participants met their recommended intake of dairy and alternatives [35]. Notwithstanding challenges in converting food intake data into estimates of nutrient intake (as discussed below), these findings suggest the need for more careful screening of current and longitudinal bone health status within the Singaporean population to help limit the high healthcare costs that can come with osteoporosis management.

The current findings are based on a cross-section large enough to ensure statistical power (in ensuring representativeness of the wider population of Singaporean children within this age range) and a data collection method originally designed to limit the potential for recall bias, as previously discussed [26,35]. The estimation of nutrient intake based on food composition tables and food records remain a major challenge in nutrition research [48]. Data on food composition was updated from the food composition tables of multiple countries and may not have truly been representative of the products consumed in Singapore.

The coding of dietary items into food groups is based on a single researcher’s (M. J. Y. C.) perspective on food categorisation. Some commonly consumed Southeast Asian foods were challenging to code as they often contain more than one type of food group in a single food item or meal. Therefore, to reduce subjectivity bias, all food items were determined according to the Health Promotion Board’s food group classification through their online nutrient analysis tool [28]. This tool was generated by a panel of experts and its use thus helped to reduce researcher bias in food coding. However, subjectivity was introduced when the original 65 food groups were aggregated into 37 food groups. This was necessary as too many food groups may increase the chance of noting unrelated combinations with undue influence (e.g., outliers of unusual foods, or when foods that are generally eaten together are broken down into detailed subgroups) [4]. There are therefore inherent challenges in defining food groups that are meaningful for both the study population and research question. The original dietary data available was duplicate 24 h recalls for each individual participant. The authors took the decision not to weight dietary data based on weekdays and weekend so as avoid overemphasising the frequency of consumption of food items consumed on the single weekday of data collection. Additional replicates of dietary intake data would have helped confirm the patterns noted within the current analysis but were not available from the original archived dataset.

Previous studies that have compared variables presented in grams and percentage of energy found that dietary patterns derived from grams of food per day were more difficult to interpret and often did not result in defining a major dietary pattern [9,38]. As such, the current approach, where weighting was based on contribution to percentage of total energy consumption appears valid but could itself bias the secondary nutrient analysis performed on the dietary clusters. Less energy-dense foods will tend to have a smaller impact on clustering but may conversely be more nutrient dense. The authors believe that because the nutrient profile of each cluster was based on the totality of all foods consumed, the chances of a finding not being truly representative of intake was minimal.

The nomenclature attached to the final pattern solutions in the cluster analysis was also subjective and was agreed on by all authors following the analysis. These terms may not directly align with other dietary patterns that have been similarly named [25]. For example, a typical “Western” dietary pattern is frequently represented by high consumption of red meat, fast foods, snacks and confectionery, and low consumption of fruits, vegetables and whole grains, and is also typically associated with a high percentage energy contribution from fat, saturated fat and sugar [25,49]. However, the “Western” terminology in the current study highlights an a posteriori cluster that included many items that could be considered to be more representative of European and North American foods.

The current findings and associated research highlight the need for more careful evaluation of dietary habit in young Singaporeans. Such future research could increase the age range of Singaporean children studied and ideally consider longitudinal data collection to better understand the relationship between health outcomes in a population based in a global food hub, where there is a high potential to see rapid, population-level changes in dietary habits over time [50].

Future studies on a posteriori dietary pattern in Singaporeans or other multiethnic populations could also reduce participant and researcher burden through use of more rapid dietary data collection tools dietary collection methods, like food frequency questionnaires. While such approaches tend to be less accurate than dietary recalls, they are usually developed with a specific population in mind [51] and existing tools that have already been developed for Singaporean adults [52] could be easily modified for use in children. An advantage of food frequency questionnaires is the ability to provide better estimates of dietary intake as compared to food records and food recalls [51]. Additional, less burdensome online technologies also show potential for improving the way in which dietary recall or food diary data are collected [53,54].

## 5. Conclusions

Cluster analysis supported definition of dietary patterns in this multi-ethnic cross-section of children. Of the three dietary patterns defined within the current study, all tended to provide an inappropriate balance of nutrients and none tended to be dominated by more prudent food item choices. Future public health efforts should continue to prioritise improving dietary habits in Singaporean children.

## Figures and Tables

**Table 1:** The percentage of energy contribution of food groups across three dietary patterns in young Singaporean children.

Name of Food Group	Total (*n* = 561)		Cluster 1 “Western” (*n* = 239; 42.6%)		Cluster 2 “Convenience” (*n* = 211; 37.6%)		Cluster 3 “Local/Hawker” (*n* = 111; 19.8%)		
	Mean	SD	Mean	SD	Mean	SD	Mean	SD	*p* *
Unsweetened beverages	0.1	1.1	0.6	3.0	0.0	0.2	0.1	0.3	0.168
Biscuits, cakes, pastries	5.2	7.3	5.6 ^a^	7.6	4.4 ^b^	5.3	5.2 ^a,b^	7.5	0.017
Breads and rolls	4.10	5.5	4.6 ^a^	5.4	3.6 ^b^	4.4	4.1 ^b^	5.6	<0.001
Other eggs	0.1	0.9	0.6	2.4	0.0	0.0	0.0	0.1	0.110
Crustaceans, shellfish and other seafoods	0.2	1.0	0.6	2.0	0.1	0.4	0.2	0.7	0.446
Processed fruits	0.2	0.9	0.6	1.9	0.4	1.1	0.1	0.5	0.657
Red meat	0.6	2.3	2.3	5.2	0.14	0.7	0.5	1.5	0.742
Cheeses	0.8	2.3	1.9	4.7	0.6	1.7	0.6	1.8	0.601
Soups (manufactured)	0.4	1.5	1.8	3.3	0.0	0.0	0.2	0.9	0.044
Stock cubes/flavourings, essence, yeast extracts	0.4	2.4	2.5	6.4	0.0	0.0	0.1	0.6	0.140
Pulses-based dishes	0.0	0.1	0.2	0.3	0.0	0.0	0.0	0.0	0.860
Red meat-based dishes	0.4	1.9	2.6	4.6	0.1	0.5	0.1	0.7	0.864
Vegetable-based dishes	1.1	2.1	1.6	4.4	0.9	1.6	1.1	1.5	0.397
Oils and fats	0.4	1.0	0.5	1.14	0.2	0.7	0.4	1.1	0.032
Leafy and non-leafy vegetables	0.9	1.7	1.5 ^a^	4.1	1.0 ^a,b^	1.0	0.8 ^b^	1.1	0.032
Breakfast cereals	1.2	3.7	1.8	3.4	4.0	7.6	0.5	1.6	0.114
Hen eggs	1.9	2.4	1.5 ^a^	1.9	2.1 ^b^	2.9	1.9 ^a^	2.4	0.009
Fresh fruits	1.9	2.6	1.8 ^a^	2.3	3.1 ^a,b^	3.8	1.6 ^b^	2.2	0.030
Ice creams	0.8	2.2	0.6	1.9	0.9	2.2	0.8	2.2	0.122
Milks	6.1	7.7	4.3 ^a^	5.6	6.5 ^b^	6.2	6.4 ^b^	8.2	0.001
Yoghurts and yoghurt drinks	0.3	1.8	0.1	0.5	1.9	4.2	0.0	0.3	0.271
Sauces, gravies, dressings and dips	0.0	0.2	0.0	0.0	0.1	0.4	0.0	0.0	0.598
Local cakes, desserts and snacks	8.3	10.4	7.2 ^a^	9.3	8.7 ^a^	8.9	8.5 ^b^	10.8	<0.001
Nuts and seeds, pulses and products	2.4	5.4	1.6 ^a^	3.0	7.2 ^b^	10.3	1.6 ^a,b^	3.4	0.016
Sugars, sweets and confectionary	2.4	4.3	1.8 ^a^	3.2	3.7 ^b^	6.7	2.2 ^a,b^	3.7	0.025
Legumes	0.1	0.5	0.3	0.9	0.4	0.9	0.0	0.1	0.145
Starchy roots, tubers, corns and stems	1.2	2.7	1.5 ^a^	3.3	2.7 ^b^	4.9	0.8 ^a,b^	1.7	0.002
Supplements	0.3	1.0	0.1	0.4	1.1	2.4	0.1	0.3	0.511
Sweetened beverages	9.0	8.0	7.4 ^a^	5.6	6.9 ^b^	5.3	9.8 ^a,b^	8.6	0.016
Buns	1.6	3.5	1.6	4.0	1.0	2.4	1.7	3.6	0.692
Grains and noodles	26.1	11.6	22.8 ^a^	11.0	21.7 ^b^	9.6	27.6 ^a^	11.7	<0.001
Fast foods	6.3	7.7	5.1 ^a^	6.2	6.1 ^b^	8.3	6.5 ^b^	7.8	<0.001
Fish and roe	2.7	4.0	2.5	3.8	1.8	2.6	3.0	4.2	0.136
White meat	5.9	6.2	5.7 ^a^	5.7	4.4 ^a^	5.9	6.2 ^b^	6.3	0.002
Herbs, spices, condiments	1.2	2.7	0.6	1.2	0.9	2.1	1.4	3.0	0.403
Fish and seafood-based dishes	1.4	2.1	1.1	1.6	1.2	2.1	1.5	2.2	0.453
White meat-based dishes	3.9	5.6	3.4	5.7	2.4	3.8	4.4	5.8	0.628

* Differences across dietary patterns and food groups were assessed using one-way ANOVA. Bonferroni correction was applied by multiplying the *p* values by the number of traits in the table. ^a,b,c^ Mean values with unlike superscript letters are significantly different between groups (*p* < 0.05) as analysed by post hoc Bonferroni test. The values in bold highlight the highest contribution to variance within the data.

**Table 2:** Estimated intake of nutrients and energy in relation to local or international recommendations

	Cluster 1 “Western” (*n* = 239; 42.6 %)		Cluster 2 “Convenience” (*n* = 211; 37.6 %)		Cluster 3 “Local/Hawker” (*n* = 111; 19.8 %)		
Dietary factor	Mean	SD	Mean	SD	Mean	SD	*p* *
Energy intake (% RDA) *	95.4 ^a^	25.9	85.8 ^b^	25.3	93.4 ^a^	25.3	<0.001
Fat (% TE)	30.7 ^a^	5.9	28.3 ^b^	5.4	31.7 ^a^	7.1	<0.001
Saturated fatty acid (% TE)	8.3 ^a^	2.9	7.6 ^b^	2.8	9.1 ^a^	3.4	<0.001
Protein (% TE)	16.5 ^b^	3.4	17.3 ^a^	3.5	15.3 ^c^	3.6	<0.001
Carbohydrate (% TE)	52.8 ^a^	7.1	54.4 ^a^	6.8	53.0 ^a^	8.0	0.047
Added sugars (% TE)	14.6 ^a^	8.4	12.9 ^a^	8.6	12.8 ^a^	8.4	0.040
Dietary fibre (% DNG) **	49.3 ^a^	29.2	53.7 ^a^	21.4	50.0 ^a^	20.4	0.083
Vitamin D (%RDA)	27.1 ^a^	67.8	25.5 ^a^	59.2	18.7 ^a^	48.7	0.479
Calcium (%RDA)	82.8 ^a^	36.1	66.3 ^b^	34.7	69.5 ^b^	38.9	<0.001
Sodium (% DNG)	135.9 ^a^	59.0	128.0 ^a^	69.2	135.6 ^a^	57.3	0.564
Iron (% RDA)	109.3 ^a^	93.4	94.1 ^a^	91.6	101.1 ^a^	90.2	0.174

RDA: Singaporean Recommended Dietary Allowances [33]; TE, total energy; DNG: American Daily Nutritional Goals [34]. * Based on physical activity level (estimated from International Physical Activity Questionnaire data from original study [26]) specific to each age and sex. ** Based on 14 g of dietary fibre per 1000 kcal of estimated energy intake. Differences across dietary patterns were assessed using one-way ANOVA. ^a,b,c^ Mean values with distinct superscript letters are significantly different from one another (*p* < 0.05) by Bonferroni post hoc test.

## Data Availability

The data presented in the analyses for this study are available on request from the corresponding author.

## Supplementary Materials

The following are available online at https://www.mdpi.com/article/10.3390/nu13041335/s1, Table S1: List of Food Groups included in cluster analysis.
