# Supplementary material for: Data-Driven Dietary Patterns, Nutrient Intake and Body Weight Status in a Cross-Section of Singaporean Children Aged 6–12 Years
Source: Nutrients. 2021 Apr 17;13(4):1335. doi: 10.3390/nu13041335 (PMC8074157; doi:10.3390/nu13041335)
Supplement: Supplementary file 1 [file nutrients-13-01335-s001.zip › nutrients-1163330-supplementary.pdf]

Supplementary Table S1. List of Food Groups included in cluster analysis.

Food Group No.	Name of Food Group
1	Sweetened beverages*
2	Unsweetened beverages*
3	Biscuits, cakes and pastries
4	Breads and rolls
5	Breakfast cereals
6	Buns (including baked, steamed, plain, and with sweet fillings)
7	Grains and noodles
8	Hen eggs
9	Other eggs (e.g., Quail eggs, duck eggs, salted and century)
10	Fast foods
11	Crustaceans, shellfish and other seafoods (e.g., squids, algae, etc)
12	Fish and roe
13	Fresh fruits
14	Processed fruits (e.g., canned, pickled, juices, etc)
15	Red meat (e.g., beef, veal, mutton, lamb)
16	White meat (e.g., poultry, duck, frog)
17	Cheeses
18	Ice creams
19	Milks
20	Yoghurts and yoghurt drinks
21	Sauces, gravies, dressings and dips
22	Herbs, spices and condiments
23	Soups (manufactured)
24	Stock cubes/ flavourings, essence, yeast extracts, etc)
25	Fish and seafood-based dishes
26	Local cakes, desserts and snacks
27	Pulses-based dishes
28	Red meat-based dishes
29	Vegetable-based dishes
30	White meat-based dishes
31	Nuts and seeds, pulses and products
32	Oils and fats
33	Sugars, sweets and confectionary
34	Leafy and non-leafy vegetables
35	Legumes (e.g., fresh beans, peas in pods)
36	Starchy roots, tubers, corns and stems
37	Supplements

*The "beverages" food group was modified to differentiate between "sweetened beverages" and "unsweetened beverages".
